# Panoramic view of key cross-talks underpinning the oral squamous cell carcinoma stemness - unearthing the future opportunities

**DOI:** 10.3389/fonc.2023.1247399

**Published:** 2023-12-19

**Authors:** Soujanya J. Vastrad, Giri Ritesh, Sowmya S. V, Ganesan Rajalekshmi Saraswathy, Dominic Augustine, Khalid J. Alzahrani, Fuad M. Alzahrani, Ibrahim F. Halawani, Heba Ashi, Mohammed Alshahrani, Reem Nabil Hassan, Hosam Ali Baeshen, Kamatchi Sundara Saravanan, Kshreeraja S. Satish, Pravallika Vutukuru, Shankargouda Patil

**Affiliations:** ^1^ Department of Pharmacy Practice, Faculty of Pharmacy, M.S. Ramaiah University of Applied Sciences, Bengaluru, India; ^2^ Department of Oral Pathology and Microbiology, Faculty of Dental Sciences, MS Ramaiah University of Applied Sciences, Bengaluru, India; ^3^ Department of Clinical Laboratory Sciences, College of Applied Medical Sciences, Taif University, Taif, Saudi Arabia; ^4^ Haematology and Immunology Department, Faculty of Medicine, Umm Al-Qura University, AI Abdeyah, Makkah, Saudi Arabia; ^5^ Department of Dental Public Health, Faculty of Dentistry, King Abdulaziz University, Jeddah, Saudi Arabia; ^6^ Department of Endodontic, Faculty of Dentistry, King Abdulaziz University, Jeddah, Saudi Arabia; ^7^ Department of Biological Sciences (Genome), Faculty of Sciences, King Abdul-Aziz University, Jeddah, Saudi Arabia; ^8^ Department of Orthodontics Faculty of Dentistry, King Abdulaziz University, Bengaluru, India; ^9^ Department of Pharmacognosy, Faculty of Pharmacy, M.S. Ramaiah University of Applied Sciences, Bengaluru, India; ^10^ College of Dental Medicine, Roseman University of Health Sciences, South Jordan, UT, United States

**Keywords:** cancer stem cell niche, miRNA, oral cancer, oral squamous cell carcinoma, oral microbiota, stemness, transcription factor, tumor microenvironment

## Abstract

The clinical management of oral cancer is often frequented with challenges that arise from relapse, recurrence, invasion and resistance towards the cornerstone chemo and radiation therapies. The recent conceptual advancement in oncology has substantiated the role of cancer stem cells (CSC) as a predominant player of these intricacies. CSC are a sub-group of tumor population with inherent adroitness to self-renew with high plasticity. During tumor evolution, the structural and functional reprogramming persuades the cancer cells to acquire stem-cell like properties, thus presenting them with higher survival abilities and treatment resistance. An appraisal on key features that govern the stemness is of prime importance to confront the current challenges encountered in oral cancer. The nurturing niche of CSC for maintaining its stemness characteristics is thought to be modulated by complex multi-layered components encompassing neoplastic cells, extracellular matrix, acellular components, circulatory vessels, various cascading signaling molecules and stromal cells. This review focuses on recapitulating both intrinsic and extrinsic mechanisms that impart the stemness. There are contemplating evidences that demonstrate the role of transcription factors (TF) in sustaining the neoplastic stem cell’s pluripotency and plasticity alongside the miRNA in regulation of crucial genes involved in the transformation of normal oral mucosa to malignancy. This review illustrates the interplay between miRNA and various known TF of oral cancer such as c-Myc, SOX, STAT, NANOG and OCT in orchestrating the stemness and resistance features. Further, the cross-talks involved in tumor micro-environment inclusive of cytokines, macrophages, extra cellular matrix, angiogenesis leading pathways and influential factors of hypoxia on tumorigenesis and CSC survival have been elucidated. Finally, external factorial influence of oral microbiome gained due to the dysbiosis is also emphasized. There are growing confirmations of the possible roles of microbiomes in the progression of oral cancer. Given this, an attempt has been made to explore the potential links including EMT and signaling pathways towards resistance and stemness. This review provides a spectrum of understanding on stemness and progression of oral cancers at various regulatory levels along with their current therapeutic knowledge. These mechanisms could be exploited for future research to expand potential treatment strategies.

## 1 Introduction

Oral Squamous Cell Carcinoma (OSCC), a subcategory of Head and Neck Cancer (HNC), arises from the mucosal lining of a wide range of anatomical regions in the oral cavity (1). World Cancer Research Fund International - 2020 ranked lip and oral cavity cancers as the 16th most prevalent and exemplified a global estimate of approximately 3,77,700 cases (2). Etiologically, Oral Cancer (OC) is linked with tobacco consumption, viral infections due to Human papillomavirus (HPV), Epstein–Barr virus (EBV) (1). Furthermore, Herpes Simplex Virus (3), and fungal infections particularly with Candida albicans (4) may also be attributed to oral tumorigenesis, however, there is a significant dearth in literature substantiating the contributory role of such infections in OC. The five-year disease-free survival rate is 80% in the case of intraoral carcinoma without metastasis, while, with regional node involvement, it reduces to 40%, and further reduces to 20% with distant metastasis. The majority of deaths occur within the first 5 years due to metastasis, which is alarming (5).

Currently, the cornerstone therapies to manage OSCC include surgical interventions, chemotherapy, Radiotherapy (RT), or a combination of these modalities. Despite recent advancements in the diagnosis and therapeutic strategies, there persist major clinical challenges owing to drug toxicity profile, tolerability issues, patient relapse, and emerging therapeutic resistance. The complexity of the situation is amplified due to the aggressive and invasive nature of the disease (6). The application of novel immunological therapies is not yet explored completely and is often accompanied by challenges to counter immune evasion (1). Foremost, the inability to target Cancer Stem Cells (CSCs) is the underlying cause of the majority of the challenges encountered in therapeutic management (7).

Conventional chemotherapy primarily aims at the apoptosis of highly proliferating cells. Although this approach is successful in eliminating the large proportion of tumor mass, the dormant CSCs manage to survive (8). These quiescent cancer cells give rise to several subclones with genetic similarities but differing functionalities. The evolutionary competition among these subclones leads to tumor heterogeneity, progression, and endurance to resist hostile conditions imparted by various therapeutic modalities (8–10).

Discrete anatomical regions harboring CSCs within the microenvironment of tumors are termed “niche” which encompasses tumor cells, endothelial and stromal cells, ECM, signaling molecules, and blood vessels (11). This region influences the regulatory elements that govern the key features and functions of CSC. This CSC niche is surmised to be modulated by intrinsic elements, such as Transcription Factors (TF) capable of cell reprogramming, and other extrinsic players of the Tumor Microenvironment (TME) (12). Moreover, TFs’ have shown their influential role on remodeling of ECM (an essential component of the CSC niche) in favor of stemness, alongside cell communication pathways (13) and cell’s behavior and responses to external cues (14). The proliferation of typical stem cells is precisely regulated by cross-talks with their physiological niche, however, CSC displays aberrant interactions and enters the mode of the abnormal self-renewal process enabling their uncontrolled growth. Moreover, the difference between the stem cell niche as observed in the normal physiology and pathological conditions influence the CSCs’ intrinsic properties such as transitioning into other cell states or being latent for a prolonged course of time or spreading to proximal and distant organs.

In malignancy, the CSC niche favors the stem cells to circumvent the stringent regulators of proliferation or programmed cell death via augmenting the recruitment of TME components such as cytokines, endothelial cells, immune cells, Mesenchymal Stem Cells (MSC), secreting growth factors and tumor-associated fibroblasts, which are crucial for nurturing the self-renewal property of CSC. In other words, this niche, preserves the core stemness features of CSC, maintains their plasticity, shields them from the immune system, and promotes their metastatic potential (12, 15, 16).

### 1.1 Stem cells in oral cancer

CSC is a small proportion of tumor mass with characteristic properties of stem cells specifically self-renewal, differentiation capacity, and pluripotency. Regardless of its minor fraction, CSCs exhibit remarkable tumorigenicity in comparison to other subgroups within the tumor mass. CSCs are surmised to manifest from either normal or progenitor stem cells through a series of transformation and reprogramming processes, or they may emerge from differentiated cells as a result of genetic and epigenetic changes during the course of malignancy development (17–19). In OC, stem cells play a major role in tumor development and progression. Thus, signifying the intricate structure of tumor mass, involving CSCs (capable of asymmetric cell division and self-renewal), transiently proliferating progenitor cells with multi-division potential, and non-contributory differentiated cells (20). Numerous markers are utilized to identify CSCs in HNC. These markers not only facilitate CSC isolation but also govern essential biological functions, including cell proliferation, invasion, self-renewal, and survival.

### 1.2 Isolation and characterization of OC stem cells

Researchers have documented the effective isolation of the CSC population in OSCC by employing a range of cell surface markers, including but not limited to CD98, CD133, CD44, and ALDH1 (21–24). In addition, certain CSCs exhibit differential expression of proteins resembling those involved in the regulation of embryonic stem cell functions, notably OCT4, NANOG, and SOX2, which serve as markers for CSCs in OSCC (25). However, it’s important to acknowledge that, there is no single, specific marker that clearly defines CSCs. Instead, multiple markers are employed in combination to identify CSCs. This underscores the inherent heterogeneity in the CSC population (26, 27).

Multiple investigations have revealed distinct CD44 expression patterns between cancer stem cells and their non-cancer stem cell counterparts across various solid tumors (28). Isolation of these CD44-positive (CD44+) CSC subgroups has been effectively achieved in HNC using flow cytometry sorting with CD44 antibodies (29, 30). CD44 is a multifaceted trans-membrane glycoprotein with its primary binding affinity towards hyaluronan. CD44 facilitates cell proliferation and survival by activating the MAPK and P13/Akt pathways (24, 31). Along the same line, other stem cell marker CD98+ subgroups exhibit high expression levels of genes associated with the cell cycle and DNA repair (21). CD133 involved in angiogenesis has been linked to a negative prognosis in OC (23). While ALDH1+ cells contribute to the development, metastasis, and resistance to therapy in HNC (32).

CSCs exhibit enhanced self-renewal capabilities when cultivated within non-adherent tumor spheres, using ultra-low binding plates to foster undifferentiated growth of these self-renewing stem cells (33). The quantity and growth patterns of tumor spheres are indicative of the self-renewal potential within a particular culture of diverse cancer cells, thus offering valuable information regarding the presence of CSC. Consequently, within the scientific community, the tumor sphere formation assay has emerged as a widely adopted method for the isolation of cancer stem cells from diverse populations of cancer cells. This method leverages the unique characteristics of CSCs for their selective separation. Notably, tumor sphere-forming cells identified in various prominent tumor types and within cultured cancer cell lines exhibit distinct attributes associated with CSCs. These characteristics are significantly more prominent when compared with the features identified in their adherent monolayer counterparts, which have traditionally been classified as non-cancer stem cells (34). Thus, tumor spheres derived from OSCC cells exhibit increased stemness features along with elevated levels of pluripotent transcription factors and stem cell markers such as Lin28, NANOG, KLF4, OCT4, SOX2, CD44, and ALDH1 respectively in comparison to their attached monolayer counterparts (35–38).

Overall, this comprehensive review embarks on multifaceted interplay which is claimed to regulate the OSCC stemness pertinent to the initiation, progression, and resistance. At the outset, the mechanism of intrinsic factors that govern the CSC microenvironment is delineated by presenting the cross-talks between TF and miRNA. Also, the contribution of extrinsic factors in the maintenance of OSCC stemness is elucidated through the interplay observed in the Extracellular Matrix (ECM), and conditions such as hypoxia, neovascularization, inflammation and infection. In addition, a precise amalgamation of avant-garde therapeutic strategies to address the real-world challenges is appraised. This compilation of complex roles may open novel avenues in the future to address the challenges and opportunities in confronting OSCC.

## 2 Intrinsic factors governing OSCC stemness

The cellular state and identity of any given cell are primarily determined by transcription profile and regulation of such profiles forms the major aspect in controlling gene activity. The intricate pattern of gene translation is led by the complex orchestration of regulatory elements such as TFs, modulators of epigenetics, and noncoding RNAs. Any mistune in the network leads to the development of malignant conditions and other diseases (39). TFs are a class of proteins that are capable of binding to specific enhancer/silencer/promoter sequences on DNA that are involved in transcription regulation. They also influence the formation of transcription initiation complexes via the enrolment of cofactors and RNA polymerases to their specific sites (40). In normal physiological conditions, the determination of stem cells to undergo differentiation or self-renewal is predominantly governed by the intrinsic regulatory mechanisms of TFs (41). These established roles along with overlapping salient features between normal and cancerous stem cells bolster the analysis of TFs in malignancy. Indeed, the repercussion of dysregulated TFs expression in cancer can be epitomized by the development of the cancer hallmarks by endowing stemness characteristics to CSC (16, 42, 43). Thus, comprehending the cellular interactions of TFs is significant for understanding the malignant progression.

In conjunction with TFs, miRNAs co-regulate the activity of the genes. It is also an intriguing fact that TFs and miRNAs influence the expression of each other. Further, the observed positive correlation between the complexity of TF’s regulation of a gene and the propensity of the same gene being governed by a miRNA strengthens the TFs-miRNA interplay. The dynamic operations of these regulators in connection with feedback and auto-regulatory loops co-ordinates various cellular events. As observed in normal stem cells, the self-renewal and pluripotency of CSCs are also regulated by the intricate TFs-miRNAs reciprocity, and understanding their interplay in the acquisition of stemness characteristics provides a better comprehension of driving factors of resistance and tumorigenicity (44).

**Figure 1:**
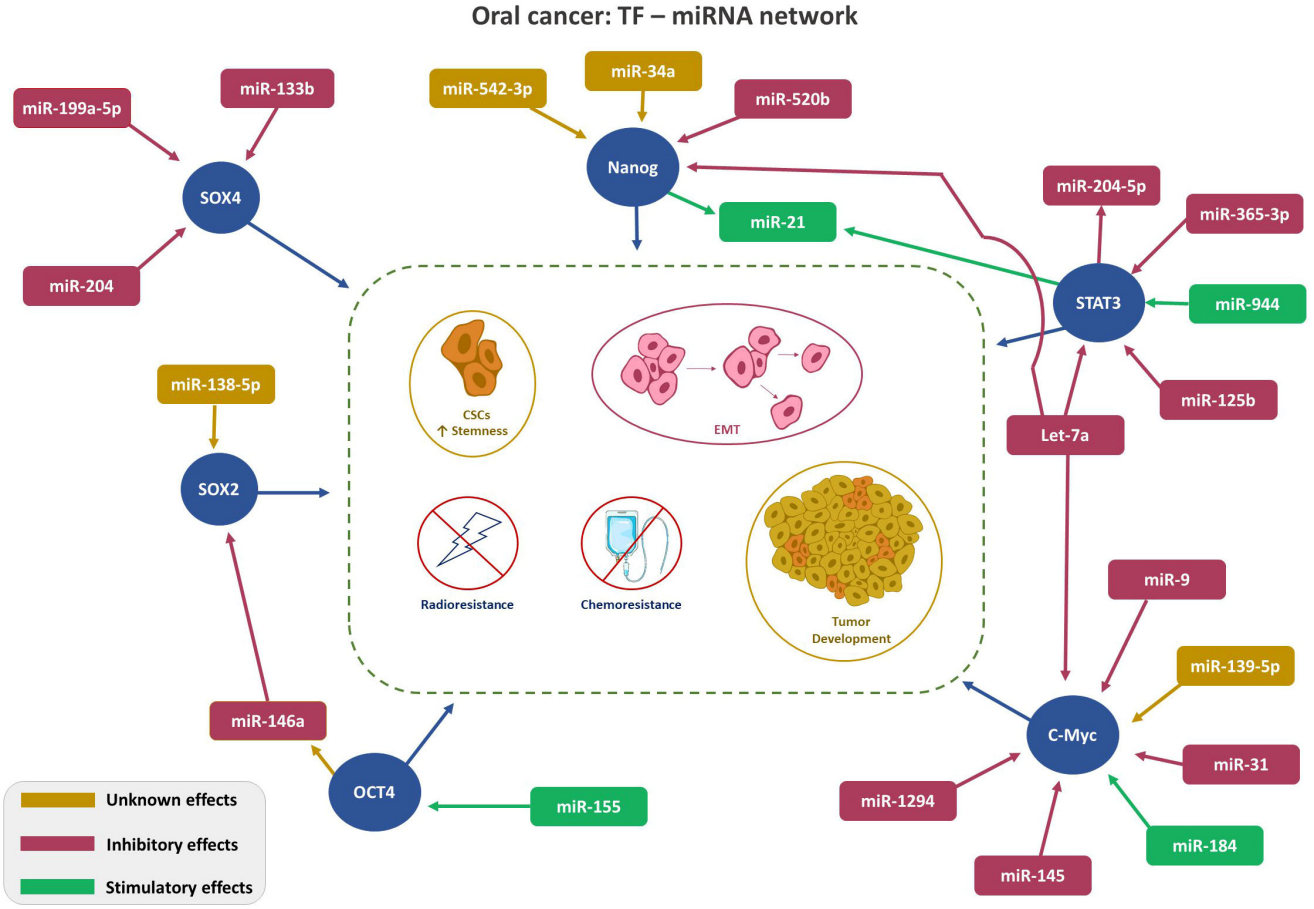
Depicts TFs-miRNA network. TF-miRNA network in Oral Cancer: Regulation of key transcription factors (SOX4, SOX2, Nanog, STAT3, and cMyc, red circle) by the indicated miRNAs. Color code (Magenta- inhibitory; Green-stimulatory; Yellow-unknown) indicates role of the miRNA on TF expression. Arrowhead: ↑ points the direction of the effect.

Despite frequent deliberations about TFs in malignant conditions, only a few are recognized to be linked with OSCC. This section presents the interplays of TFs: NANOG, OCT, c-Myc, SOX and STAT with miRNA in OC. **Figure 1** depicts TFs-miRNA network.

### 2.1 NANOG-miRNA network in OSCC

NANOG is an important element of the TF ensemble with a characteristic homeodomain that is expressed during early human development (45). It plays a role in the self-renewal and preservation of ground-state pluripotency of embryonic stem cells. Diverse expression patterns of NANOG in embryogenesis, for instance, the elevated expression in embryonic stem cells and reduced expression in primitive endodermal cells are largely governed by an activin/SMAD signaling pathway.

NANOG is silenced in mature and differentiated cells. The mechanism of NANOG pertinent to maintenance of pluripotency of undifferentiated cells is not clearly comprehended, however, it is presumed to repress the genes that lead to differentiation. Also, this TF is believed to activate the OCT4 gene among others, which helps in the upkeep of an undifferentiated state resulting in over-proliferation and rapid spread (46–48).

Of interest, OSCC studies have shown higher expression of NANOG in the CSC subpopulation (49). Additionally, higher levels of NANOG and OCT4 have been correlated with advanced cancer stages and lower survival rates among patients in both OSCC and Pulmonary adenocarcinoma (35, 50). Some of the acknowledged factors governing NANOG expression include activation of STAT3, Hedgehog signaling pathways, and micro-environmental conditions favoring hypoxia (51–53).

In this section, the impinging roles of various miRNAs in regulating NANOG expression in OSCC are conferred.

Patel S et al. (54), investigated the characteristics and underlying mechanisms of CD44+ CSC-like OSCC subpopulation in view of their distinct capabilities to grow anchorage-independent along with a high degree of self-renewal potential. The results indicated a positive association between CD44 and NANOG expressions and speculated miR-542-3p and miR-34a as their prominent regulators.

Another study in HNC, inclusive of OSCC cell lines by Lu YC et al. (55), established the diverse role of miR-520b in regulating the anti-malignant properties such as augmenting cell susceptibility to radiation and chemotherapy, restricting the cell movement and limiting the formation of CSCs. It was also demonstrated that the formation of spheroid cells and expression of NANOG were suppressed, thus indicating the prospective governing role of miR-520b in OSCC cancer stemness.

In addition, the investigation led by Cheng-Chia Yu et al. (56), demonstrated a significant reduction in let-7a expression, while, the levels of NANOG/OCT4 was observed to be increased in HNC tissues as compared to adjacent non-cancerous tissues. This inverse association was significantly observed in recurrent and nodal metastatic HNC status as compared to their expression in the parental tumors.

The meticulous work of Bourguignon LY et al. (57), untangled the possible role of NANGO/STAT3 signaling in promoting chemo-resistance using HSC-3, a human OSCC cell line. The authors illustrated that the binding of hyaluronan, a matrix glycosaminoglycan ligand to CD44 promoted the formation and nuclear localization of the NANGO/STAT3 complex. The resultant complex enhanced the expression of the miR-21 gene and the production of mature miR-21. This showed a downstream inhibitory function on protein expression involved in chemotherapy-induced programmed cell death (PDCD4). Thereby contributing to chemo-resistance.

### 2.2 OCT4-miRNA network in OSCC

OCT4 is observed to be expressed in early embryonic stages. Within the early embryo, this TF is expressed in totipotent and pluripotent cells of the blastocyst inner cell mass and epiblast, respectively, which possess the ability to differentiate into all types of the embryo proper (58). In recent times, researchers have observed OCT4 expression in tumor cells and not that of normal somatic tissues (59). Additionally, studies have demonstrated the close association of OCT4, with SOX2 and NANOG in orchestrating cellular reprogramming. In the context of OSCC, the presence of OCT4 has been associated with a significant increase in tumor transformation, tumorigenicity, invasion, and metastasis. Thus, it may be speculated that OCT4 may have an extended role in the regulation of epithelial-mesenchymal transition (EMT) and its potential significance as a marker for CSC (60).

Ghuwalewala S et al. (61), studied the impact of miR-146a in CD44high and CD24low groups within OSCC cells and primary HNC tumors. An increase in the levels of miR-146a was observed, which enhanced the stemness characters by stimulating CD44high and CD24low groups. Further mechanistic analysis revealed stabilization of β-catenin by miR-146a with a simultaneous reduction in E-cadherin and CD24. It was also demonstrated that the aberrant expression of this miRNA had an influential role on OCT4.

Another study by Juan-Song et al. (62), examined the relative relation between OCT4 and miR-155. The results revealed an increased expression of miR-155 in OSCC tumor samples concomitantly with that of OCT4.

### 2.3 c-Myc-miRNA network in OSCC

c-Myc serves as a classical TF as well as a global regulator of chromatin structure through histone acetylation (63). This TF is versatile in regulating an array of cellular processes inclusive of protein biosynthesis, metabolism, cell cycle regulation, and cell adhesion in normal physiological conditions (64). Whilst, compelling evidence from research studies demonstrates an upsurge of c-Myc expression in 80% of the OSCC (65) and self-renewal of tumor stem cells (66). Moreover, overexpression of this TF is correlated with poor prognosis (67). This section of the review highlights the shared relation observed in the c-Myc-miRNA axis pertinent to OSCC.

Numerous studies have elucidated the downregulation of miR-145 in different cancers and this miRNA is reported to exhibit a down-regulatory effect on c-Myc. Subsequent examination of its putative function in OC revealed that the silencing of miR-145 as an attributing factor to evasion of tumor suppression and thus leading to tumorigenesis.

This fact is further confirmed by Shao Y et al. (68), wherein, restoration of miR-145 in Tca8113 cells exhibited substantial growth suppression by impeding cell proliferation, while re-expression of the same was observed to promote cell cycle arrest and apoptosis. Thus, miR-145’s tumor suppressive role is mediated via its negative feedback on c-Myc. These findings are linear with other cancer types, for instance, colon and non-small cell lung cancer.

miR-1294 is another regulatory mediator that targets c-Myc. Wang Z et al. (69), revealed a negatively correlation of miR-1294 with c-Myc levels. Consequently, reduced levels of miR-1294 is linked to unfavorable prognosis of OSCC.

miR-let-7a, has been confirmed to control diverse signaling cascades in tumors. The tumor-suppressive characteristics of this miRNA is widely quoted in earlier studies and the downregulation of which could enhance the proliferation and migration of tumor cells. Luo C et al. (70), showcased miR-let-7a downregulation in OSCC along with overexpression of c-Myc.

The function of miR-9/9-3p in the advancement of malignancy is often debatable owing to its tumor promoter or suppressor role in solid tumors. Elevated expression of miR-9 in primary breast cancer cells has endowed metastatic potential (71), conversely, miR-9-3p overexpression is correlated with reduced rates of cell proliferation of hepatocellular carcinoma cells (72). Online with the latter, in OSCC, miR-9 overexpression diminished cell proliferation along with metastatic potential and colony-forming ability. Subsequent cell stage analysis revealed G0/G1 suppression (73). Similar to miR-9, miR-184 also demonstrates controversial roles in malignancy. Ryan DG et al. (74), demonstrated the expression of miR-184 in the epithelial cells of the germinative zone using an animal model. However, this has been less explored in human malignancy conditions, the first report on aberrant expression in OSCC was reported by Wong TS et al. (75), Their study revealed the antiapoptotic and proliferative functions of miR-184 in tongue squamous cell carcinoma. The roles of both miR-9 and miR-184 in malignant conditions were attributed partly due to inhibitory and stimulatory effects on c-Myc expression, respectively.

The potential targets of miR-31 were studied by Jung JE et al. (76), in Drosophila melanogaster and OSCC cell line models. The study confirmed the association of miR-31 in the maintenance of human Wntless mRNA, which is a key regulator of Wnt signaling. Furthermore, downregulation of two important transcriptional targets of Wnt signaling, namely cyclin D1 and c-Myc were observed. This suggests the possible role of miR-31 in governing the cell cycle and proliferation of OSCC cells.

In another preclinical animal model, a reduced miR-139-5p was showcased to augment proliferation and invasion of OSCC via WNT-responsive elements such as c-Myc, cyclin D1, Bcl-2, and CXCR4 (77).

### 2.4 STAT-miRNA network in OSCC

STAT belongs to a family of dormant cytoplasmic TFs, which upon activation in response to various extracellular polypeptides regulate gene expression (78). In pathological conditions, oncoproteins induce a persistent transformation of latent STAT to its active state, via tyrosine kinase signaling pathways. This dysregulation leads to uncontrolled growth mediated via abnormal expression of Bcl-xl, cyclin D1, c-Myc, and others, thus causing neoplasm. In conjunction with this evidence, EGFR-mediated constitutive activation of STAT3 leads to tumor advancement and apoptotic dysregulation in squamous cell carcinoma (79, 80).

Another notable causative factor for angiogenesis could be related to a perpetual active state of STAT3 followed by an upsurge in vascular endothelial growth factor stimulation. Taken together, recent findings provide validating facts explaining the STAT3’s role in cancer and the abrogation of its constitutive active state provides a new targeted strategy in cancer therapy. This section primarily focuses on the regulatory axis of STAT and miRNAs by direct or indirect involvement of regulatory components such as JAK1, SOCS1, and PIAS3 (81). STAT3 in its constitutive active and overexpressed state has found its role in disease progression, resistance and as well as enhancing the stem cell features by modulating the transcription of various downstream target genes, for example, extracellular vesicles derived from CSCs promoted the stemness and chemoresistance via PI3K/mTOR/STAT3 signaling in OSCC (82).

Several studies comprehending the dysregulation patterns of miRNA in malignant conditions have concluded the overexpression of miR-21 (14). The study conducted by Zhou X et al. (83), on human OSCC tissues observed a co-expression of miR-21 and STAT3. Furthermore, mechanistic analysis of suppression of miR-21 and STAT3 co-expression by employing STAT3 inhibitor revealed an upsurge in tumor suppressive phosphatase and tissue inhibitor of metalloproteinase expression. Thus asserting the oncogenesis mediated via STAT3/miR-21 pathway.

Zhuang Z et al. (84), carried out the most detailed clinical and ***in vivo*** analysis of miR-204-5p and elucidated its onco-suppressive role in HNC inclusive of OSCC. This study correlated a loss of miR-204-5p with the stimulation of Epithelial to Mesenchymal Transition (EMT) and STAT signaling pathways which govern tumor initiation and metastasis. miR-204 directly targets the signaling proteins involved in stemness such as SNAI2, SUZ12, HDAC1, and JAK2. A reduction in miR-204-5p expression exerts a stimulatory effect on the formation of the SNAI2/PRC2/HDAC1 repressor complex, resulting in a further reduction of miR-204-5p levels. Additionally, in the course of the transcriptional process, STAT3 displayed interaction with SNAI2/PRC2/HDAC1 repressor unit to suppress miR-204-5p. Thus, resulting in a feedback loop governing the stemness.

Chang SM et al. (85), proposed STAT3 as a possible target of miR-125b and delineated the shared mechanism of MALAT1 a long non-coding RNA, miR-125b and STAT3 axis in OSCC condition. This research revealed the oncogenic role of the aforesaid axis being attributed to the over-expressed state of MALAT1 alongside upregulated STAT3 and repressed miR-125b. Thus, an inverse association was noticed between miR-125b and STAT3.

The ambivalent role of miR-944 has been observed in various malignant conditions. For instance, in cervical cancer miR-944 has a tumorigenic nature by targeting HECW2 (HECT domain ligase W2) and S100P binding protein (86). Conversely, in colorectal cancer it exhibits a tumor-suppressive role via inhibiting cell growth and progression by targeting GATA binding protein (87). In OSCC, Peng HY et al. (88), studied the complex mechanism of miR-944/CISH/STAT3 axis in an inflammatory microenvironment. The results revealed an upsurge in miR-944 leading to a concurrent downregulation of CISH, which is an important immune response mediator. Thus, directing the oncogenic role of miR-944 in OSCC. It was also evident that the miR-944-mediated silencing of CISH enhanced the pro-inflammatory gene expression directed by STAT3 activation.

Let-7 family is a prominent member of matured miRNA family. In general, they are tumor suppressive by abrogating stemness characters, such as differentiation and regeneration of CSC. Accordingly, their reduced expression in malignant conditions explains the disease progression (89). In OSCC, Li X et al. (90), elucidated the inhibitory role of let-7a on STAT3.

Huang WC et al. (91), focused on untangling the miR-365-3p/EHF/keratin 16-axis mechanism to discern its role in OSCC metastasis and drug resistance. Findings established an intrinsic link between keratin protein KRT16 with integrin β5 subunit and c-Met which resulted in the activation of Src/STAT3 signaling in promoting cell invasion and metastasis. Furthermore, to this, miR-365-3p was found to target ETS homologous factor by modulating KRT16 and opposed the aforesaid oncogenic signaling. This study evinced a decreased miR-365-3p expression and elaborated on the stimulation of STAT3-mediated discrete signaling pathways and regulation of stemness characters of cancer cells.

### 2.5 SOX-miRNA network in OSCC

SOX modulates the DNA-protein interaction owing to its high-mobility group domain and is fundamentally involved in various cellular signaling events of neoplastic conditions. In OSCC, overexpression of SOX2 has been shown to enhance invasiveness and its ability to grow independently of anchorage, thus, signifying its involvement in fostering stem cell-like traits (92).

The study by Wei D et al. (93), revealed the tumor suppressive property of miR-199A-5p in OSCC. It primarily suppressed the oncogene SOX4 and thus restrained cell mobility and invasion. Further, the mechanistic analysis revealed its influence on the EMT process largely by reducing the E-cadherin expression and concurrently increasing the levels of other cell migratory proteins such as N-cadherin, vimentin, and fibronectin.

Shi Z et al. (94), explored the role of miR-146a in OSCC through a functional analysis which revealed a proportionate decline in miR-146a expression with the advancement of tumor stages. Subsequent evaluation demonstrated that the experimental elevation of miR-146a expression downregulated the SOX2. Overall miR-146a was found to have metastatic suppressive properties.

Chia Yu CC et al. (95), noticed decreased expression of miR-204, particularly in OSCC-derived ALDH1+ CSCs. Overexpression of this miRNA in the ***in vitro*** experimental settings displayed repression of cancer stemness and tumor growth. This functionality was attributed to its binding affinity towards slug and SOX4 which resulted in reduced expression of these TFs in OSCC stem cells.

Zhuang Z et al. (96), analyzed the differential gene expression in OSCC tissue samples. The results defined TP63 (particularly ΔNp63) as the candidate gene that was linked with tumor development. This candidate gene was functionally associated with the suppression of miR-138-5p. The aforesaid cross-talk mechanism with ΔNp63 was considered to indirectly modulate the genes associated with stemness such as SOX2, CD44, NOTCH1, and KLF4. The study conducted by Yang J et al. (97), observed a reduced expression of miR-133b in OSCC and confirmed its inverse association with SOX4.

## 3 Extrinsic factors governing OSCC stemness

**Figure 2:**
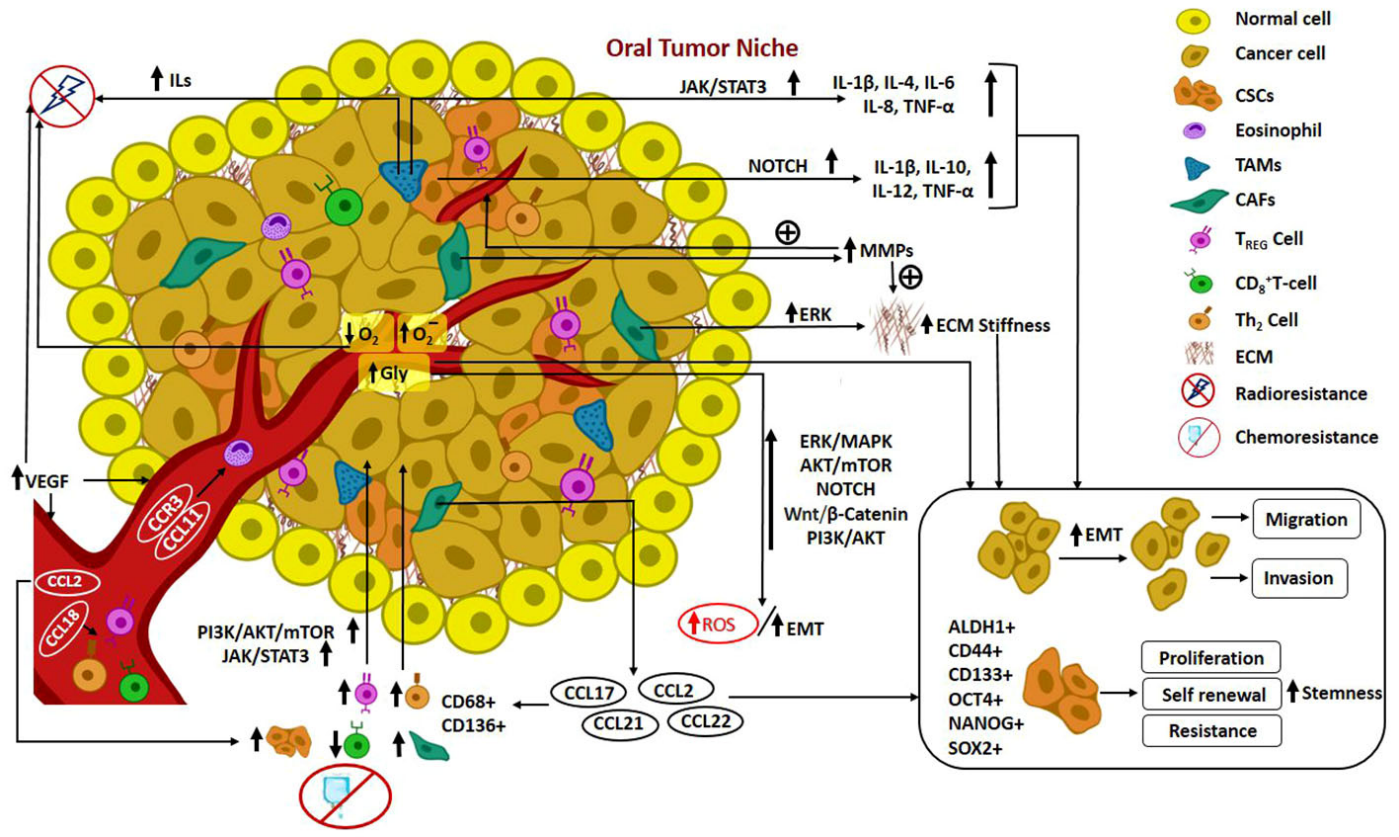
Illustrates the mediators and pathways governing oral tumorigenesis and stemness in the TME. Cross talks in Oral CSC niche: Recruitment of fibroblasts, growth factors, determinants of EMT, inflammatory and immune components from TME; hypoxic features; angiogenesis mechanism; mediators and pathways governing oral tumorigenesis and stemness in the TME.

In recent times, the notion of cancer ecology has emerged, viewing cancer as an evolutionary ecological process. The growth of cancer cells is contingent upon interactions with elements in the TME, involving a mutual exchange of substances that facilitate mutualism and co-evolution. Furthermore, the progression of malignancy is regarded as an ecological invasion (98). Cross talks occurring in the CSC niche pertinent to the elements recruited from TME such as fibroblasts, growth factors, determinants of EMT, inflammatory and immune components, hypoxic features and angiogenesis mechanisms are delineated in detail. **Figure 2**. illustrates the mediators and pathways governing oral tumorigenesis and stemness in the TME.

### 3.1 Cross-talks involving ECM components

ECM is a non-cellular network of cross-linked macromolecules comprising glycoproteins, collagens, and proteoglycans which forms a crucial supramolecular scaffold. It offers numerous valuable cues that influence tumor development and metastasis (99).

#### 3.1.1 Cancer-associated fibroblasts

CAFs are vital stromal cells present in the TME recruited by tumor-secreted factors. They are involved in the synthesis and remodeling of ECM components, which in turn affects tumor progression (100). CAFs expressing Lysyl Oxidase, a copper-dependent amine oxidase (101), and α-Smooth Muscle Actin (a-SMA), were found to augment proliferation, migration, invasion, and EMT processes in OSCC cells. The underlying interplay was characterized by the upregulation of vimentin and N-cadherin, down expression of E-cadherin, and cross-linking of collagen leading to increased matrix stiffness causing ECM remodeling via phosphorylation of the FAK pathway. This pattern of expression also contributed to increasing tumor differentiation and poor patient prognosis (102). Besides the FAK pathway, matrix stiffness and transcription of tumor cells are linked to elevated AMPK levels and were further stabilized by Integrins (ITGAV or Integrin aV, to be more specific), showcasing ITGAV-FAK-AMPK-Autophagy signaling axis as a worthy target for future therapeutic approaches. Thus, it is clear that fibroblasts induce stromal autophagy, creating a protumorigenic niche for cancer development (103).

#### 3.1.2 Growth-regulated oncogene alpha

A study analyzed the role of GRO-α derived from oral submucous fibrosis-associated fibroblasts in OSCC development. GRO-α, a member of the CXC family was found to be upregulated in dysplastic oral keratinocytes and promoted the proliferation, migration, and anchorage-dependent growth of these precancerous cells by enhancing the EGFR/ERK signaling and F-actin rearrangement. Additionally, cancer stemness was established by increased expression of NANOG. This infers that GRO-α facilitates oral malignant transformation attributing to oral tumorigenesis (104). Another study observed elevated levels of GRO-α and tumor-promoting interleukins corresponding to the expression of angiopoietin-like protein. This can in turn induce CAF-like phenotypes within the stromal fibroblasts of OSCC cells and increase the levels of CAF markers, a-SMA and FAP, making this a potential quarry for targeted therapies in the future (105).

#### 3.1.3 Transforming growth factor β

TGFβ is a cytokine secreted within the ECM. This intricately controls a plethora of biological mechanisms during carcinogenesis and metastasis. TGFβ mediates antiproliferative properties and acts as a tumor suppressor in the course of early tumorigenesis, while, at advanced stages, it acts as a tumor promoter cytokine assisting in metastatic progression via an autocrine TGFβ loop (106). TGFβ-1, via phosphorylation of SMAD2, was found to stimulate MMP-2 and MMP-9 with the aid of the MT1-MMP/MMP2 axis. Activation of MMP9 could potentially contribute to the proteolysis of the matrix which could further support the invasion of OSCC cells (107). TGFβ generates slower-cycling squamous cell carcinoma stem cells and also mediates tumor invasiveness, cell dissemination, and aberrant differentiation of these cells by a non-genetic paradigm. These slow-cycling cells have further shown to develop resistance towards traditional chemotherapy agent cisplatin, by preventing apoptosis of these malignant cells. Further, TGFβ expression can markedly enhance glutathione metabolism by activating p21, which stabilizes NRF2 and enhances not only chemoresistance but also metastasis and survival of these CSCs. These findings open new avenues for design and development of chemotherapeutics that might circumvent drug resistance (108).

#### 3.1.4 Factors influencing EMT

EMT is a vital course in tumorigenesis where a polarized epithelial cell can undergo a myriad of biochemical changes to transform into a mesenchymal cell phenotype. This transformation results in increased invasiveness, migratory capacity, resistance towards apoptosis, and generation of ECM elements within the tumor (109). Several TFs influence the EMT process, for instance, SOX2 was found to enhance EMT traits along with stemness properties such as invasiveness, anchorage-independent growth, and xenotransplantation tumorigenicity among CD44 and ALDH1-rich OSCC cells. SOX2 expression was also responsible for increasing resistance towards radiation therapy and cisplatin, making SOX2 a good therapeutic target (92). Conflictingly, CD44 positivity was found to decrease linearly with increasing cancer cell differentiation, leading to an increased Tumor Budding Activity (TBA) and a smaller Cell Nest Size (CNS), two key features driving the EMT process. This altered CD44 expression was in favor of high OSCC aggressiveness and unfavorable epithelial cell transition (110). Similarly, the ectopic expression of SOX8 was found to initiate EMT and stemness properties. This was characterized by elevated BMI1, SOX2, OCT4, and ABCG2 markers within cisplatin-resistant OSCC cells by means of the FZD7-mediated Wnt/β-catenin pathway, significantly contributing to poor prognosis and increased chemo-resistance (111). NFATc3, a member of TF family was found to be upregulated in ADLH1-rich OSCC CSCs. Herein, NFATc3 demonstrated binding with OCT4, a stemness factor, that harbored the self-renewal properties and tumor sphere formation along with increased migration capacity. Additionally, NFATc3 expression conferred enhanced cisplatin resistance in these OSCC cells. This study unveiled the NFATc3-OCT4 axis as a novel pathway underlying the CSC/EMT features, oral tumorigenesis, and chemo-resistance. Prospective drug research can unearth potential therapeutic targets underlying the aforesaid interplays (112).

### 3.2 Cross-talks involving inflammatory cytokines and immune complexes

Cytokines are tumor cell secretomes involved in aberrant cell differentiation, proliferation, metastasis, angiogenesis, and survival alongside promoting interactions between the cells. They assist in the recruitment of multiple immune cells, thereby triggering immune-mediated cancer cell neutralization. Tumor-Associated Macrophages (TAMs), mast cells, dentritic cells and T-cells are some of the immune cells which are vital components of the tumor microenvironment dictating tumorigenesis, cancer progression and stemness. Mast cells and dentritic cells have been involved in modulating the microenvironment (TAM and lymphocytic recruitment) and helping neoplastic cells evaded the immune system. However, TAMs are one of the major immune cells involved actively in promoting tumor development and progression (113). This section sheds light on some of the cross-talks of key pro-tumor cytokines with the OSCC niche.

#### 3.2.1 Interleukins

IL-6 and IL-8 are the two predominant interleukins expressed in HNC (114). A research carried out by Hsu PC et al. (115), correlated the overexpression of IL-6 and IL-8 with the promotion of invasiveness of OC cells via phosphorylation of the STAT3 signaling pathway. Additionally, these cytokines were considered to initiate the EMT process featured by reduced E-cadherin and augmented vimentin expression within these cells. Overexpression of IL-8 can result in enhanced cancer stemness and tumor aggressiveness. This was in agreement with the study conducted by Peng CY et al. (116),. This study unveiled that Let-7c was significantly downregulated in OSCC cells. This in turn enhanced the IL-8 overexpression, attributing to the self-renewal capacity of these tumors. Additionally, this pattern of expression was also responsible for increased resistance towards chemo-radiation with cisplatin. Lee CR et al. (117), assessed the differential expression of around 25 cytokines in oral tumor spheres and linked their interdependency with the Jumonji domain-containing protein 6 (JMJD6) which is a histone arginine demethylase protein. Amongst the studied cytokines, IL-4 was found to be significantly increased and directly correlated to JMJD6 modulation, leading to its increased expression. This high JMJD6 expression mediates oral carcinogenesis, anchorage tumor growth, and migration, and augmented CSC properties of self-renewal and colony formation. JMJD6-regulated CSC phenotype was established by upregulation of genes associated with CSC such as FGF4, Gli1, Gli3, IL-4, Lin28A, Lin28B, OCT4, Zeb1, and Zeb2. These results revealed that JMJD6 regulated IL-4 expression via binding to IL-4 promoter in CSC, signifying a novel CSC governing process involving the JMJD6-IL4 axis. Intriguingly, increased JMJD6 expression also exhibited resistance towards traditional chemotherapy agents such as doxorubicin, methotrexate, and etoposide.

#### 3.2.2 Tumor necrosis factor alpha

TNF-α is an inflammatory cytokine which takes part in the maintenance of various cellular signaling processes (118). TNF-α is one of the most abundant pro-inflammatory cytokines followed by CXCL-8 in the oral MSC secretome, which could potentially mediate tumor development (119). Tumor associated macrophages (TAMs) play a vital role in the production of TNF-α which can be associated with increased stemness in OC (120). Similarly, CSC expressing high levels of CD44 were found to significantly elevate TNF-α secretion along with other inflammatory cytokines such as Interleukins (IL-1beta, IL-10, IL-12) and angiogenic factors (Angiopoietin-1 & 2, VEGF, and bFGF) owing to increased oral tumor stemness (121). TNF- α was reported to increase the proliferation capacity by constantly elevating the key stemness TFs such as KLF4, Lin28, NANOG, and OCT4 in HPV-immortalized oral keratinocytes. On a similar note, this cytokine maintains CSC properties such as expression of surface markers (CD44high/CD24low), increased migration, self-renewal capacity, and anchorage-independent growth, which could promote the malignant growth of OC. A novel activation of the TNFα/miR-203/miR-200c axis was evinced to promote the aforesaid CSC characteristics. Herein, the microRNAs: miR-203 and miR-200c were downregulated, in turn triggering the Notch signaling pathway. In the context of therapeutic failure, TNF-α expression was interrelated with increased resistance towards cisplatin and RT (122).

#### 3.2.3 Chemokines

Chemokines are subtypes of cytokines with ambivalent potential as they are part of the tumor inflammatory process, involved in neoplasia alongside recruitment of various tumor-associated immune cells (123). Multiple chemokines and their receptors interact with each other and the TME components to mediate various clinical facets of cancer development, progression, and stemness (124, 125).

In general, chemokines mediate the infiltration of CD8+T cells in the TME thereby, displaying an anti-tumor effect. In HNC and OSCC, the following chemokines: CCL5, CCR5, CCR2, CXCR3, and CXCL9 were found to be downregulated. This repression is negatively linked with YKT6 gene overexpression, which is presumed to be due to modifications in the DNA methylation levels and copy number variation in this gene. This differential YKT6 gene expression was reported to result in reduced infiltration of CD8+T-cell in the tumor niche. Furthermore, patients with copy number amplification of YKT6 gene demonstrated a reduced CD4+T cell and B cell infiltration (specifically in HPV-positive cohorts), reflecting that this genetic aberration may be linked with the remodeling of the immune microenvironment in HNC/OSCC. Immune dysregulation in the TME of this sort could potentially favor malignant progression, recurrence of tumors, and poor patient prognosis (126). CXCL8 is another pro-inflammatory cytokine that was found to be a vital component in the propagation of Oral Cancer Cells (OCC) (119). CCL2 is a major player in proliferation, migration, invasion, and tumor growth, via phosphorylation of NF-κB and STAT3 pathways. Production of CCL2 was majorly from CAF and its high expression among OCC was the reason for the CAF-OCC cross-talk. CCL2 upregulation was responsible for ROS production and vice-versa, involving the PI3K/Akt/mTOR pathway, leading to elevated levels of cell cycle proteins (cyclin D, cyclin E, and CDK4) and ultimately, oral carcinogenesis (127). CAF-expressed chemokines such as CCL17 and CCL22 were both found to upregulate T_reg_ infiltration within the OSCC microenvironment, favoring an immunocompromised tumor background (128). CCL18 is another chemokine that is found to be upregulated in OSCC cells and is responsible for the promotion of EMT via overexpression of Slug protein (an EMT-associated TF). In addition, cancer stemness was enhanced by expression of OCT4 and Bmi-1 alongside tumor sphere formation within ALDHhigh+ and CD133-expressed cancer cells. All of these cascades were ascribed to mTOR pathway activation (129).

CCL21 and CCR7 were upregulated in OSCC cells and their interaction was found to induce EMT via loss of E-cadherin and gain of vimentin & N-cadherin, further enhancing the invasion, proliferation, and migration. Concurrently, CSC-related markers such as ALDH1A1, BMI, CD44, CD133, and OCT4 were upregulated, directly suggesting that the CCL21/CCR7 axis was responsible for increased OSCC stemness and chemo-resistance towards cisplatin, elucidated via activation of the JAK2/STAT3 pathway (130). Certain eotaxins (selective eosinophil chemoattractant), for instance, CCL11 (Eotaxin-1) showed enhanced interaction with CCR3 resulting in increased eosinophil infiltration, leading to a tumor-associated tissue eosinophilia condition in the OSCC niche. However, the functions of eosinophils in the oral tumor background needs to be further investigated (131, 132). Another chemokine, CCL22 was found to be overexpressed in tongue cancers. Its increased interaction with CCR4 led to enhanced recruitment of TAMs (mostly, M2-type), T_reg_, and Th2 cells with a suppressed CD8+T-cell infiltration. This altered immune behavior exhibited an increased proliferative index and poor overall survival rate (133).

#### 3.2.4 Tumor-associated macrophages

Infiltration of TAMs in the tumor background has not only been correlated with tumor progression (134), but has also contributed to cancer stemness and poor treatment outcomes. TAMs are typically maintained in an M2-polarized state and are also involved in the remodeling of the ECM and immunosuppression in the TME (135).

Hsieh CY **et al.** showcased the infiltration of TAM into the TME. These macrophages were responsible for the secretion of various interleukins, IL-1β being the predominant one. Increased IL-1β secretion within the TME is believed to upregulate ICAM1 expression, which enhances cancer stemness. IL-1β was also reported to activate superoxide dismutase 2 and inhibit catalase, thereby modulating the ROS levels intracellularly and further activating ICAM1 expression. Upon ICAM1 activation, mesenchymal markers such as fibronectin, N-cadherin, and vimentin were observed to be elevated whereas, epithelial marker (E-cadherin) was suppressed, reinforcing the increased EMT features. Further, the IL-1β-SOD2/CAT-ICAM1 pathway is exemplified to contribute to chemoresistance towards docetaxel (136). TAM markers, CD68 and CD163 were highly expressed in OSCC samples in comparison to normal oral mucosa and dysplastic cell samples. Expression of stem cell markers such as ALDH1, CD44, and SOX2 was directly related to CD68 and CD163 positivity. The presence of these stem cell markers directly affected the tumor stage and pathological grading but the expression of the TAM markers did not have any influence on these tumor characters. However, expression of CD68 and CD163 were remarkably related to the aggressive behavior of OSCC, including nodal involvement. Significant expression of CD163 was linked with an unfavorable overall survival in patients, and thereby serves as a potential diagnostic and prognostic marker in OSCC patients (137).

M1-like TAMs have also been shown to uphold the malignant progression of OSCC cells by modulating the CSC characteristics. An important correlation amongst M1-related markers (CD68, CD80 and CD86) and elevated levels of EMT-related markers (Ki67, CD10, CDH2, TWIST1, VIM, and SNAI1) was identified. Additionally, MME and MMP14, the CSC markers, were also found to be sharply increased, indicating the regulation of the EMT/CSC process of OSCC cells. Similarly, IL-6 was markedly secreted from these macrophages and exhibited a significant correlation with CD80 and CD86, thus regulating M1-like TAM functions such as migration, invasion, and colony as well as microsphere formation via stimulation of the JAK/STAT3 pathway. Additionally, STAT3 activation enhanced the transcription of Thrombospondin-1 (THBS1) in OSCC cells, which promoted a positive feedback mechanism among M1-like TAMs and OSCC cells displaying mesenchymal/stem-like phenotype (138, 139).

### 3.3 Cross-talks in hypoxic and glycolytic-laden microenvironment

The canonical hypothesis on the effect of hypoxia on malignant cells is thought to morphologically constrain them. Surprisingly, they adapt and transform into a more aggressive state by intensifying neoplastic drivers. The hypoxic environment is much more prevalent in rapidly proliferating neoplastic cells due to the growing metabolic demands. However, their adaptive “Warburg effect” stratagem enables them to survive in the most hostile oxygen-deprived conditions (140). The Cancer Genome Atlas dataset revealed that OSCC, a subset of HNSCC, is the most hypoxic type of cancer among various other malignancies. OSCC manifests with substantial necrotic areas, in which CSC resides in acidic and hypoxic conditions (141, 142). There are also reports to indicate that hypoxia fosters cell migration and CSC stemness (143). The adaptive mechanism involves the fine interplay mediated by Hypoxia Inducible Factor (HIF), unfolded protein reaction, mTOR signalling, and Autophagy. Dong W et al. (144), focused on comprehending the putative oncogenic role of the Special AT-rich sequence-binding protein 2 gene (SATB2) in OSCC under hypoxic conditions. An increase in the assemblage of autophagosomes, the transformation of microtubule-associated protein light chain – LC3-I to LC3-II, and an upsurge in the Beclin-1 expression with stemness markers such as OCT4, SOX2, NANOG were observed. Additionally, silencing of SATB2 demonstrated suppression of colony-forming ability led by hypoxia. These findings provide a vital linkage between stemness with hypoxia in OSCC. In line with this, an experimental study by Chatterjee R et al. (145), concluded the concomitant upregulations of the Sonic Hedgehog pathway (Shh-Gli-1) and the preponderance of hypoxia in OSCC.

Another study by Duan Y et al. (146), showed that hypoxia augments Bcl-2/Twist1 interaction by inducing and amplifying Bcl-2 binding to Twist1, thereby facilitating the EMT process. Further, it was observed that the rate of nuclear translocation of these factors was higher in tumor cells under hypoxic conditions. This would synergistically promote the transcription of downstream target genes resulting in a sequence of alterations in cell phenotype remodeling, migration, invasion, and tumor growth. Further, Marconi GD et al. (147), showcased the interaction between certain transcriptional genes, c-Myc, and HIF. This c-Myc-HIF interlink collaborated with oncogenic signaling pathways such as Akt/mTOR, Notch signaling and ERK/MAPK. This resulted in altered cell cycle, cell metabolism, ribosome biogenesis, and genomic stability in oncogenesis. The observed pathophysiological relationships corroborate the stemness prevailing in hypoxic conditions.

Expression of HIF-1α has been linked to hypoxia-induced oxidative stress mediated via ROS generation in OSCC cells. Further, the expression of 4-Hydroxynonenal, a product of lipid peroxidation, was also evinced. Increased oxidative stress was considered to promote the EMT process, facilitating tumor migration through enhanced phosphorylated ERK activity. Additionally, there was elevated B-catenin colocalization in the OSCC cells’ nuclei. Another striking finding in this study was the contribution of the upregulated Shh/Gli-1 signaling axis and survivin overexpression in further mediating the EMT process, acquiring stemness properties associated with CD133 and ALDH1 overexpression, maintaining the CSC phenotype, and facilitating ROS production in these hypoxic OSCC cells (116). Hypoxia enhances the expression of a transcriptional protein namely SATB2 which promotes OSCC tumor development and stemness. SATB2 overexpression was further linked to hypoxia-induced autophagy characterized by increased LC3-I, LC3-II, and Beclin-I levels. Additionally, SATB2 influenced the OCT4, SOX2, and NANOG mediated stemness, invasive and migratory properties of these cells. Overall, this study concluded that the knockdown of SATB2 could be a promising therapeutic approach to downregulate these stemness and tumorigenic properties of OSSC cells, by inducing cell cycle arrest (G0/G1) and apoptosis even under hypoxic conditions (144). Conversely, ROS-mediated ER stress was claimed to be beneficial in inducing apoptosis within OSCC cells (148).

Glycolysis provides the necessary energy required by cancer cells to undergo proliferation and regulate apoptosis (149). Per2, a vital gene involved in glucose metabolism in various cancers (including OSCC), was found to enhance glycolysis. Downregulation of this gene increased the levels of key glycolytic rate-limiting enzymes such as hexokinase, pyruvate kinase, and lactate dehydrogenase and thereby, inhibited apoptosis and facilitated the proliferation of OSCC cells via the PI3K/Akt pathway (150). Another study identified the role of enhanced HIF-1α and mTOR expression in initiating glucose metabolism within OSCC cells. Additionally, GLUT1 and hexokinase 2 levels were found to be significantly increased in these cells, indicating a potential involvement of the PI3K/Akt/HIF-1α pathway in oral tumorigenesis by promotion of glycolysis and gluconeogenesis (151). Overexpression of GLUT1 not only correlates with glycolysis but also with enhanced hypoxia and angiogenesis in the preliminary stages of OSCC development. Co-expression of GLUT1 and OCT 3/4 is regulated by stem cells in these infant phases of OC development and has a direct correlation with unfavorable tumor differentiation and patient prognosis (152). Glycolysis was also responsible for facilitating the EMT in OSCC cells which was characterized by elevated vimentin & snail and reduced E-cadherin expression. Stemness was also maintained due to enhanced CD44, NANOG, and CD133 expressions. These findings are suggestive of the pro-tumorigenic roles of glycolysis in oral tumor development, migration, and poor patient prognosis (153).

### 3.4 Cross-talks influencing angiogenesis

Marconi GD et al. (147), demonstrated the significant expressions of Bcl-2, c-Jun, c-Myc, ERK 1/2, pERK1/2, HIF-1α, MMP-9, and VEGF proteins among untreated OSCC cell samples in comparison to cells treated with doxorubicin. The aforesaid proteins are allied with angiogenesis, hypoxia, inflammation, and enhanced migration, invasion and survival. Among the suspected proteins, a vital interplay identified between Myc-HIF-1α could offer a conducive microenvironment for the growth and stemness of these OSCC cells. Incorporating doxorubicin was considered to downregulate the pathways activated by c-Myc, which might reduce these tumorigenic features and present c-Myc as a potential therapeutic target. THBS1 was found to stimulate various Matrix Metalloproteinases (MMPs) such as MMP3, MMP9, MMP11, and MMP13 partly via the integrin signaling and coordinate OC invasion. Additionally, MMP-9 was associated with angiogenesis in OC (154, 155). Another study found that OSCC-associated vascular endothelial cells showed exaggerated CD44 expression which was associated with increased proliferation and angiogenesis. CD44 expression was in turn responsible for the elevation of ECM proteins such as TGFβ and MMP9, which further enhanced the fusion of these endothelial cells with ECM components to promote tumor neovascularization. Even though the exact mechanism underlying CD44 mediated angiogenesis is not well established, it can definitely be taken into account that CD44 positivity can serve as a good diagnostic biomarker to potentially direct the initiation of anti-angiogenic therapies (156). Aberrant VEGF and CD44 expression in OSCC cells could also contribute to radioresistance. The relationship between these two markers could also pave a path towards the possible mechanism dictating this radioresistance. Hence, combining VEGF or CD44 inhibitors with RT would be an acceptable option for achieving a better treatment outcome (157).

## 4 The impact of oral microbiota on OSCC tumorigenesis

**Figure 3:**
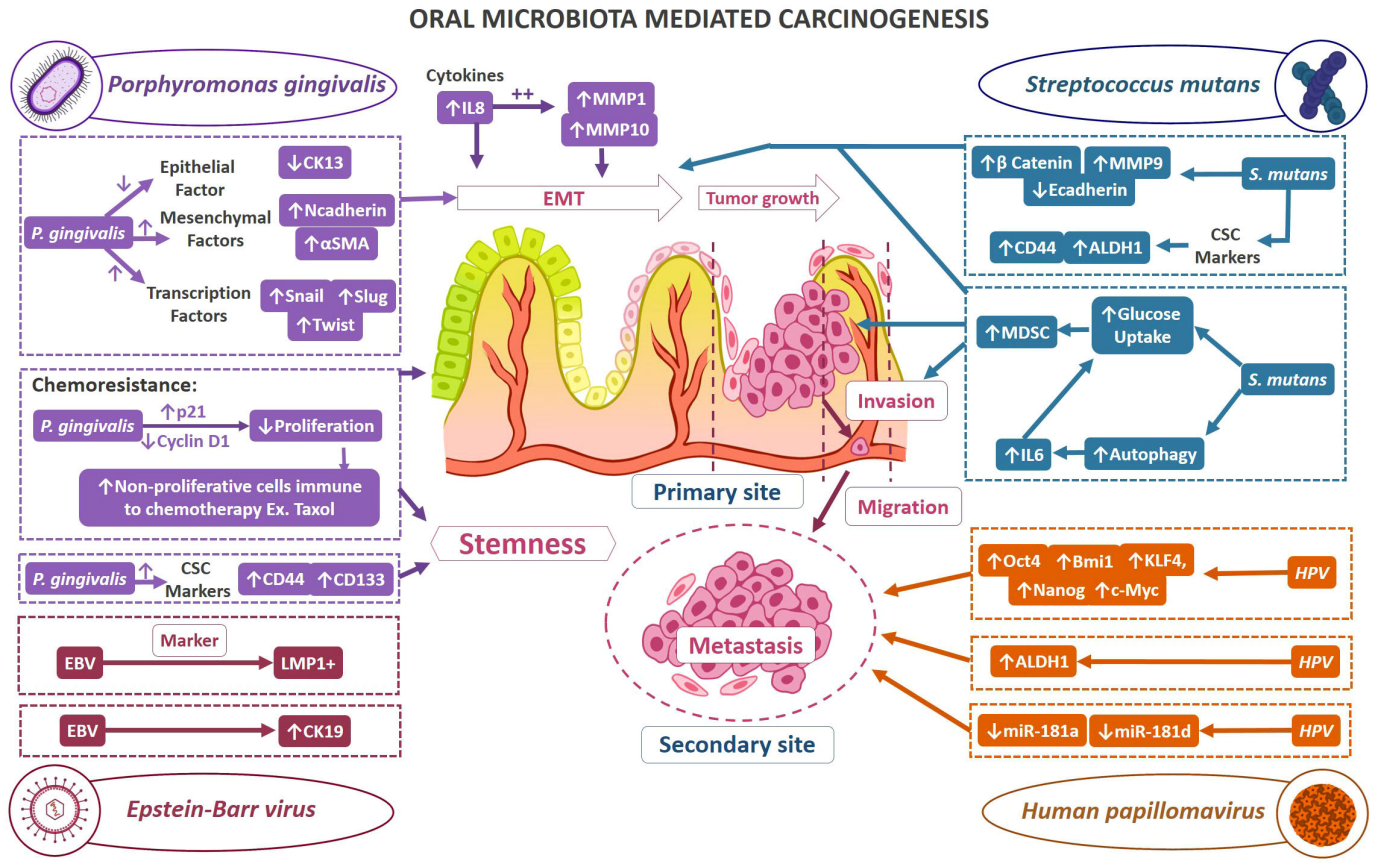
Demonstrates the mechanisms of selected oral microbes in oral tumorigenesis and stemness. The possible mechanisms by which oral bacteria contribute to oral carcinogenesis and stemness; Color code of mechanism representation: *Porphyromonas gingivalis* mechanism (purple color), *Streptococcus mutans* (teal blue), *Human papillomavirus* (orange), Epstein-Barr virus (magenta).

Current research findings have acknowledged the involvement of various infectious agents in tumorigenesis. In healthy status, the microbiome shares a sophisticated homeostatic balance with their human hosts, a phenomenon that is increasingly referred to as normobiosis. However, any perturbation to this eclectic microbial composition could lead to dysbiosis which is often related to the transition from healthy to diseased conditions (158). For instance, dental caries and periodontitis are considered as microbial dysbiosis-associated diseases (159). Furthermore, it has been demonstrated that disrupted oral microbiota could mediate the fulfillment of the major hallmarks of cancer. Thus, of late within the scientific community, there is a heightened emphasis on comprehending the contribution of oral microbes in the tumor development. Viruses such as EBV, hepatitis B & C virus, human T-cell leukemia virus-1, Kaposi sarcoma-associated herpesvirus, Merkel Cell polyomavirus, and Simian 40 virus have been correlated in neoplasia (160). Amongst bacteria, it is a well-established fact that *Helicobacter pylori* has a significant relation with various gastrointestinal cancers (161). Addressing the roadblocks in decoding the multifaceted mechanisms of OSCC is also correlated with microbiomes. Earlier studies utilizing culture and biochemical characterization have demonstrated an abundance of C. albicans, aerobes, and anaerobes on the tumor surfaces in comparison to the healthy mucosal surface (162). The impinging role of microbes in OSCC progression is attained by virtue of distorting the host cell proliferation and apoptosis equilibrium, immune dysfunction, and affecting the host metabolism. Some of the acknowledged carcinogenic mechanisms observed in infectious conditions are DNA alterations caused by bacterial toxins as well as due to the reactive oxygen and nitrogen intermediates produced by the host. When these damages surpass the cell restoration, they may cause mutational changes and thus leading to cancerous conditions. β-catenin signal transduction is another frequently observed target of microbes resulting in the upregulation of genes that normally take part in cell proliferation. Inflammatory conditions engendered due to the infection, mediate the signaling pathways involving NF- κb and STAT3 (163). This section mainly involves examining the influence of the oral microbiome in the acquiescence of stemness in OSCC. **Figure 3**. demonstrates the mechanisms of selected oral microbes in oral tumorigenesis and stemness.

### 4.1 Influence of *Porphyromonas gingivalis* in OSCC

*P. gingivalis*, a gram-negative oral anaerobe magnifies chronic periodontitis to an aggressive OSCC (164). The aforementioned fact was reinstated by Sayehmiri F et al. (165), through their meta-analysis revealing that the existence of microbe increases the risk of advancement of carcinoma by more than 1.36 times. Interference with tissue integrity followed by disruption of the host cell’s immune response such as instigating cell proliferation, and triggering chronic inflammation alongside suppressing apoptosis and cometabolite generation are the key onco-pathological mechanisms mediated via *P. gingivalis* (166).

This section appraises the migratory and invasive potential of prolonged exposure of *P. gingivalis* on OSCC cells. Ha NH et al. (167), observed EMT subsequent to repetitive infection of OCC by *P. gingivalis*, which was characterized by reduced expression of epithelial marker, Cytokeratin 13; and upsurge of mesenchymal markers such as N-cadherin and α-SMA, alongside TFs such as snail, slug, and twist. OSCC cells infected with *P. gingivalis* also exhibited a higher degree of migratory potential. Further, an intriguing mechanism of chemo-resistance conferred by *P. gingivalis* on OSCC cells was evinced. Herein, *P. gingivalis* slowed down the proliferation of OSCC cells which accounted to higher levels of cell cycle inhibitor, p21 and reduced levels of cell cycle progression molecule, cyclin D1. Since, non-proliferative state cells are insensitive to a number of chemotherapeutic agents, *P. gingivalis*-infected OSCC cells exhibited chemoresistance with paclitaxel treatment. In agreement with the fact that EMT and chemoresistance correlate with cancer stemness, this study demonstrated the overexpression of CSC markers such as CD44 and CD133 with repeated *P. gingivalis* infection. Subsequent analysis of cytokine profile in *P. gingivalis* infected OSCC cells was carried out to capture the association of EMT with cytokines. This revealed the overexpression of IL-8. Later, the involvement of MMPs in cancer migration and invasion was examined, which demonstrated a substantial upregulation of MMP-1 and MMP-10 in *P. gingivalis* infected OSCC cells. Further, upon studying the influence of IL-8 on MMP, it was observed that IL-8 selectively increased the release of MMP-1 and MMP-10. By infecting OSCC with *P. gingivalis*, this study simulated the TME of OSCC cohorts with chronic periodontitis and showcased the outcome of periodontitis in the development of OC at the molecular status.

### 4.2 Influence of *Streptococcus mutans* in OSCC

Given the fact that oral microbiota demonstrate a key association with the advancement of OC, the increased scrutiny of a wide range of microbes disclosed the carcinogenic potential of Streptococcus species.

For instance, presence of *S. anginosus* was observed in human oral and pharyngeal cancer biopsy samples (168). This observation was reinforced by several research studies (168, 169) signifying this species as a promising non-invasive biomarker to aid in early detection of oropharyngeal cancer (170). In case of *S. mitis*, there were controversial reports on its association with OSCC (171, 172).

This section focuses on the tumorigenic potential of *S. mutans* in OSCC. Tsai MS et al. (173), investigated the microbiome diversity in oral biofilms of OSCC patients and healthy controls to delineate the potential link between the enriched organisms with OSCC. The study revealed a higher species richness and a substantial difference in the overall microbiome composition in OSCC biofilms as compared to control. Subsequent linear discriminant analysis captured *S. mutans* to be differentially enriched with more abundancy in OSCC condition, amongst the observed pool of Streptococcus microbiome. Additionally, DESeq2 analysis demonstrated a substantial difference in *S. mutans* uncovering its role in OSCC. For further precision, qPCR displayed an ample amount of 16S rDNA pertinent to *S. mutans* in cancer samples signifying the high frequency of incidence and abundance of this species in carcinoma. Moreover, the levels of this microbe of interest were found to be enhanced in the biofilms of patients with gross tumor than those devoid of the same. Post this, DNA extraction from the OSCC samples coupled with qPCR revealed the significance of *S. mutans* in locally advanced carcinoma compared to early stage, indicating its association with OSCC prognosis. Similarly, the greater prevalence of *S.mutans* in cancer tissues was also associated with a poor disease-control contributing towards tumor aggressiveness.

Consecutive experimental research involving 4NQO- induced oral-tongue cancer in mouse correlated the *S. mutans* infection with enhanced glucose uptake by the lesions, Myeloid-Derived Suppressor Cell (MDSC) recruitment and risk of developing invasive carcinoma.

Moreover, ***in vitro*** wound healing assays utilizing *S. mutans*-infected human OSCC cells demonstrated an enhanced EMT-linked features such as increased β-catenin and MMP-9 levels, lowered E-cadherin expression, and higher levels of CSC markers such as CD44 and ALDH1 along with noticeably increased invasive potential. Additionally, the microbial infection is also reported to potentiate lung metastasis.

*S. mutans* infection-induced autophagy caused IL6 expression which is evinced by attenuation of the later post treatment with 3-methyladenine, an autophagy inhibitor. Additionally, ***in vitro*** IL6 suppression reduced the expression of EMT markers in OSCC cells infected with *S. mutans* in addition to weakening cell invasion.

The relationship between tumor growth and IL6 signaling studied in orthotopic xenograft model demonstrated that *S. mutans* infection greatly increased glucose uptake, which is connected to accelerated tumor growth and EMT alterations.

Conclusively, it is acknowledged that *S. mutans* infection contributes to OC development and progression with its potential role in enhancing the levels of IL-6, EMT-related, and CSC-related proteins besides, MDSC recruitment.

### 4.3 Influence of human papillomavirus in OSCC

HPV, especially HPV-16 infection is reported as a significant etiological factor contributing to Oropharyngeal Squamous Cell Carcinoma (OPSCC) in developed countries. Moreover, the American Joint Committee on Cancer staging system in their recent revision distinguished HPV-positive and HPV-negative OPSCC as discrete entities possessing unique molecular characteristics and tumor features, with the former demonstrating a better prognosis. Deriving hints from the above established role of HPV in OPSCC, the current section focuses on elucidating its influence on the disease biology of OSCC (174).

Lee SH et al. (175), investigated the virulence augmenting role of HPV-16 in the HPV-negative OSCC cell lines. Initially, HPV-16 whole genome was transfected in OSCC cell lines (UM6, SCC105, UM10b, SCC66) devoid of HPV to assess the promotion of malignant phenotype. This revealed that HPV-16 E6 and E7 were expressed in the transfected cell lines. Viral transfection imparted a robust anchorage-independent growth ability, colony-forming efficiency, and malignant histomorphology with invasive features in OSCC cells. Later, xenograft tumor assay in nude mice displayed a dynamic increase in tumorigenicity in HPV-transfected animals. Subsequent tumor sphere formation assay showcased the self-renewal property of HPV-negative OSCC on exposure to high-risk HPV. This underscores the presence of stemness features mediated through elevated expression of c-Myc, Bmi1, KLF4, OCT4, and NANOG and associated self-renewal capacity. Besides, flow cytometry revealed a dramatic increase in the ALDH1 population, a crucial CSC marker. Transwell migration assay and matrigel invasion assay displayed a noteworthy enhancement in migration and invasion properties respectively, accentuating the stemness characters in the HPV-16 transfected group.

Further, miRNA expression profiling of HPV-16 transfected OSCC cells revealed a consistent downexpression of miR-181a and miR-181d. These results demonstrated that HPV16 increased tumor development and CSC phenotype among HPV-negative OSCC cells by transcriptionally subduing miR-181a/d. Novel therapies may be aimed at restoring the expression of miR-181a/d to abrogate HPV-induced oral carcinogenesis.

### 4.4 Influence of Epstein-Barr virus in OSCC

There are compelling evidences to showcase the association between EBV and malignant conditions, however, their carcinogenic role is still under investigation. EBV-positive individuals are often associated with presence of infection in pharyngeal lymphoid tissues which serve as a crucial source of the virus (176). Kis A et al. (177), found that 73.8% of OSCC patient samples were supportive for presence of Latent Membrane Protein-1 (LMP-1), a marker of most EBV-related malignancies, compared to the controls (19.1%). The study by Jiang R et al. (178), is based on the fact that the relationship between EBV and OC is much sporadic and its role as a carcinogen is still ambiguous. The study examined the expression level of Cytokeratin 19 (CK19) an intermediate epithelial filament protein that serves as an epithelial stem cell marker. It was demonstrated that expression levels of CK19 were remarkably higher in EBV-infected dysplastic epithelium in comparison to negative ones and the same was observed in the case of infected and non-infected OSCC conditions. These findings of higher transcript levels of CK19 in EBV-positive conditions within similar grade of dysplasia or malignant differentiation is highly intriguing.

## 5 Stratagems in targeting pathways involved in OSCC stemness – a therapeutic perspective

Surgery, chemotherapy and radiation therapy have always been the main pillars of oral cancer therapy since the early 20th century, with the former being practiced since the medieval times. Over the last two decades, targeted therapies (such as cetuximab or afatinib) and immunotherapies (such as pembrolizumab or nivolumab) have been incorporated into the standard treatment guidelines given the improvement in patient survival (179). However, resistance towards traditional therapies has always been a big challenge in cancer treatment, in addition to poor affordability towards novel agents given their varied availability and high cost. As mentioned earlier, OSCC cells are programmed to enhanced tumorigenesis, progression and stemness via activation of multiple cell signaling cascades such as JAK/STAT3, ERK/MAPK, PI3K/Akt/mTOR, Wnt/β-catenin, Notch etc. These conduits can serve as excellent therapeutic targets going ahead which can possibly overcome such therapy related issues.

This section sheds light on the possible therapeutic approaches that aim to downregulate some of these pathways involved in oral cancer development and stemness. **Table 1**. summarizes the drug candidates and their possible mechanisms that target the above-mentioned signaling pathways to reduce oral carcinogenesis, progression and stemness features.

**Table 1:** Clinical targets.

THERAPY CANDIDATE	TARGET PATHWAY/SIGNALING INVOLVED IN CANCER STEMNESS	MECHANISM
**MPT0B098 [181,182]**	JAK2/STAT3	↑ Apoptosis ↑ Cell cycle arrest ↑ Effectiveness of 5-FU and Cisplatin
	TGF-β/SMAD	↓ Hypoxia-induced EMT
**Tyrphostin AG490 [183]**	JAK2/STAT3	↓ Tumor Proliferation ↓ Tumor Migration ↓ EMT
**Honokiol [184]**	JAK2/STAT3 AKT ERK	↓ Tumor Migration ↓ Apoptosis ↓ Survival/Proliferation of CSC
**JAK Inhibitors** **(Ruxolitinib, Tofacitinib)** **[202]**	JAK/STAT	↓ Tumor Aggressiveness ↓ OSCC Stemness
**OTX008 [185,186]**	MAPK	↓ Tumor Proliferation
	AKT	↓ Tumor hypoxia ↑ Efficacy of radiation and penetration of chemotherapy
**U0126 [188]**	MEK	↓ Tumor Migration
	TGFβ/ERK/Snail	↓ EMT
**LY294002 [187]**	PI3K/AKT	↓ Tumor Migration
**Disulfiram [188,148]**	TGFβ/ERK/Snail	↓ EMT
	Not specified	↑ UPR and ER stress ↓ Tumor Proliferation ↑ Apoptosis
**PI-828 [189]**	PI3K/AKT/mTOR	↓ Tumor Proliferation and Colony Formation ↑ Cell cycle arrest (G0/G1 phase) ↑ Apoptosis and Autophagy ↓ Tumor migration, invasion and angiogenesis
**PI-103 [189]**		↓ Tumor Proliferation and Colony Formation ↑ Cell cycle arrest (S phase) ↑ Apoptosis and Autophagy ↓ Tumor migration, invasion and angiogenesis
**PX-866 [189]**		↓ Tumor Proliferation and Colony Formation ↑ Cell cycle arrest (G2/M phase) ↑ Apoptosis and Autophagy ↓ Tumor migration, invasion and angiogenesis
**BKM120 (Buparlisib) [190]**		↓ Tumor Proliferation ↑ Radiosensitivity
**BYL719 (Alpelisib) [190]**		↓ Tumor Proliferation ↑ Radiosensitivity
**AZD2014 (Vistusertib) [190]**		↓ Tumor Proliferation ↑ Radiosensitivity
**Niclosamide [193]**	Wnt/β-catenin	↓ Self-renewal capacity and CSC enrichment EMT ECM remodeling ↓ Tumor migration, invasion ↑ Apoptosis ↑ Chemosensitization towards Cisplatin
**ICG-001 [194,195]**		↓ EMT ↓ Migration of CSCs
**Celecoxib [199,200]**	NOTCH	↓ Tumor Differentiation/Grade ↓ EMT ↓ Migration of CSCs and recruitment of MDSCs
**DAPT [201,196]**		↓ Tumor Proliferation ↓ Stemness properties when combined with Docetaxel, Cisplatin and 5-FU
**γ-secretase Inhibitors (LY411575, RO4929097)** **[202]**		↓ Notch activation ↓ Tumor Aggressiveness ↓ OSCC Stemness

### 5.1 JAK/STAT3 signaling

JAK/STAT pathway is a well-known mechanism involved in mediating vital downstream events of cellular communication and functions. This pathway constitutes a rapid membrane-to-nucleus signaling module and induces the expression of important mediators of cancer and inflammation (180). As described earlier, the JAK/STAT3 pathway can be involved in promoting stemness properties and chemoresistance within OSCC cells via the CCL21/CCR7 axis, making this cascade a potential therapeutic target (130). MPT0B098 is a novel microtubule inhibitor that has been shown to inhibit the JAK2/STAT3 pathway in multiple cancers. In OSCC cells, this agent was found to induce apoptosis and cell cycle arrest by promoting the accumulation of suppressor of cytokine signaling-3 protein, further facilitating the ubiquitination and degradation of JAK2 and TYK2, promoting to the loss of STAT3 activity. Hindrance of STAT3 activity led to sensitization of OSCC cells towards MPT0B098 cytotoxicity, confirming that STAT3 is a vital mediator of drug resistance in oral carcinogenesis. Moreover, conventional drugs like 5-Fluorouracil (5-FU) and cisplatin showed significant augmentation of OSCC cells when combined with MPT0B098, rather than using this novel inhibitor alone or just the 5-FU/Cisplatin dual chemotherapy, making this novel combination therapy a promising therapeutic option (181). Additionally, MPT0B098 was also found to reduce hypoxia-induced EMT due to the destabilization of HIF-1α by downregulating vimentin and N-cadherin and by the partial expression of EMT-activating TFs such as SNAI2/Slug and Twist. MPT0B098 was further able to suppress hypoxia-induced EMT via inhibition of TGF-β/Smad signaling and by interfering with FAK-mediated actin cytoskeleton rearrangement (182). Tyrphostin AG490, a selective inhibitor of JAK2/STAT3 was found to abrogate the proliferation, migration, and EMT process of OSCC via inhibition of the NIR1-CCL18 axis, by ultimately downregulating the JAK2/STAT3 pathway (183). Honokiol, a herbal constituent that possesses various anti-tumor and anti-angiogenesis properties, was found to inhibit tumor sphere formation in oral CSC-like OSCC cells by impeding the JAK2/STAT3 pathway activity. This herbal remedy was also responsible for inducing apoptosis within these cells via the inactivation of the anti-apoptotic Bcl-2 protein and conversely increasing the expression of pro-apoptotic Bax proteins. Further, migration of these OSCC cells was also reduced due to a substantial downregulation in the IL-6 levels, in turn suppressing the JAK2/STAT3 signaling (184).

### 5.2 ERK/MAPK and PI3K/Akt/mTor signaling

Honokiol was found to further eliminate the CSC-like OSCC properties by downregulating the Akt and ERK signaling (184). OTX008, a selective Galectin-1 (Gal-1) inhibitor, was found to abolish the protumorigenic properties of Gal-1 in OSCC cells. This agent was found to reduce the OSCC cell viability in a dose-dependent manner via induction of the MAPK pathway, following the FOS gene regulation (185). OTX008 has been found to reduce oral tumorigenesis with minimal toxicity and increase the overall tumor oxygenation by normalizing vascularization, in comparison to Bevacizumab. Certain cell lines (SQ20B) that exhibit p53 mutation enables them resistant towards chemoradiation. Consecutive mutations within EGFR further regulates rich Akt signaling associated with enhanced tumor development. Given that OTX008 can reduce tumor hypoxia, the oxygen-abundant microenvironment can serve as a good medium to increase radiation-induced oxidative killing of the OSCC cells. Administering chemotherapy in this course of OTX008-mediated oxygenation window can also potentiate drug penetration or diffusion and overall tumor coverage (186). U0126, a MEK protein inhibitor, and LY294002, a PI3K/Akt inhibitor, were found to reverse the EGF-induced migration and reduced MMP-9 levels in the OSCC cells but did not affect the phosphorylated Akt and ERK protein levels (187). However, in another study, U0126 and disulfiram were found to reverse EMT by reducing vimentin levels, elevating E-cadherin expression, and reducing p-ERK protein levels via inhibiting TGFβ/ERK/Snail signaling pathway, irrespective of SMAD4 expression. Overall, this study indicates that disulfiram was found to reduce invasion and migration (***in vitro***) while it inhibited tumor growth and metastasis (***in vivo***) in these OSCC samples, paving a path for more studies in the future for clinical use (188). PI-828, PI-103, and PX-866 are PI3K inhibitors that were found to significantly abrogate OCC proliferation and colony formation. All three agents were found to induce cell-cycle arrest: PI-828 in G0/G1 phase, PX-866 in the G2/M phase and S-phase, and PI-103 in S-phase. There was an induction of apoptosis, autophagy, and reduction in the migratory, angiogenic, and invasive properties of these OSCC cells upon exposure to these agents. These were characterized by the downregulation of VEGF, Bcl-2, NF-kB, COX-2, P110α, Pan-Akt, total mTOR, and p-mTOR, predominantly in cells treated with PI-103. To add on, pNF-ĸB/p65 was found to be accumulated more within the cytoplasm of the treatment-sensitive cells than in the nucleus, revealing a reduced protein translocation state and its degradation. These results suggest a promising role of these PI3K inhibitors in OSCC patients by disrupting the PI3K/Akt/mTOR signaling (189). BKM120 (Buparlisib) and BYL719 (Alpelisib) are two other PI3K inhibitors. Buparlisib and Alpelisib were both found to inhibit OC growth in a dose-dependent manner but only Buparlisib showed superior activity against radioresistant OCC in comparison to Alpelisib. When RT was combined with both these PI3K inhibitors, there was significant radiosensitization achieved in all OSCC cells when compared to RT exposure alone, which was confirmed by reduced colony formation. Further, when AZD2014 (Vistusertib), a competitive mTOR inhibitor was combined with RT and either BKM120 or BYL719, there was profound inhibition among the radioresistant OSCC cells when compared to the previous dual-combinations and even with AZD2014 with RT. These triple combination therapy options can dysregulate the PI3K/Akt/mTOR pathway, resulting in an intense anti-tumor effect against radioresistant OSCC cells (190).

Hypoxia or altered glycosylation can activate the Unfolded Protein Response (UPR), a homeostatic mechanism in protein synthesis that predisposes OSCC cells to increased ER stress (191). In this context, disulfiram was shown to facilitate apoptosis and decrease the proliferation of OSCC cells by activating the UPR and ER stress (148).

### 5.3 Wnt/β-catenin signaling

The Wnt/β-catenin pathway in multiple cancers is known to facilitate cell proliferation, differentiation and stem cell self-renewal capacity which also regulates response towards various therapies (192)). Niclosamide, an anthelmintic drug, reduced the self-renewal ability of ALDH+ OSCC cells. This agent was also responsible for deregulating the EMT process, ECM remodeling, migration, and invasion of these malignant cells, which were characterized by elevated E-cadherin and TIMP2 levels while, vimentin, Snail, c-myc, MMP2, and MMP9 were reduced in a dose-dependent manner. The anti-cancer effect of niclosamide was mediated by the downregulation of the Wnt/β-catenin signaling, which downregulated β-catenin, Cyclin D1, DVL2, and p-GSK3β proteins in the ALDH+ treated cells. Stemness features were further suppressed which was demonstrated by reduced SOX2, OCT4, and NANOG expression. Most importantly, niclosamide reduced the cisplatin-induced OC stem cell enrichment, enhanced the sensitization of cisplatin among ALDH+ OSCC cells, and induced apoptosis within these cells (193). ICG-001, a small molecule inhibitor, interferes with OSCC cell growth by inhibiting the β-catenin and cAMP-responsive element binding (CREB)-binding protein (CBP) activity by increasing their cytosolic localization from the nucleus. Genes dictating Wnt/β-catenin signaling (CCND2, CDK1, DKK1, LEF1, SKP2, and WNT5B) and cell survival/proliferation (BIRC5, CCNE1, CCNE2, CCNB1, CCNB2, CDKN3, and CDCA7) were found to be significantly downregulated in the treated OSCC cells. Treatment with ICG-001 drastically reduced the EMT process within these OSCC cells, exhibiting a higher E-cadherin and lower vimentin expression. This agent selectively targeted stem cells expressing CD24, CD29, and CD44 and eliminated their metastatic potential by downregulating the β-catenin/CBP activity. Notably, patients who show the presence of the aforementioned signature genes were confirmed to have better activity with this anti-cancer therapy, directly correlating to overall survival (194, 195).

### 5.4 Notch signaling

Notch signaling pathway was also responsible for enhancing the OC stemness characterized by ALDH1, CD44, CD133, SOX2, and Slug expression. DAPT, a gamma-secretase inhibitor when combined with traditional agents such as docetaxel, cisplatin, and 5-FU, showed a significant reduction of these CSC properties via blockade of the Notch1 pathway (196).

Notch signaling has been found to initiate OSCC carcinogenesis by increasing the proliferation, migration, and stemness properties of these cells by communicating with various components of the TME (197, 198). This section presents the role of Celecoxib (CXB) in dysregulating Jagged-1/Notch pathway and thereby conferring a robust anti-tumor effect.

CXB is known to regulate the Cyclooxygenase-2 (COX-2) levels involved in mediating various tumor-related immune cells such as MDSC, TAM and Tumor-Endothelial Cells in the oral TME. The selective COX-2 inhibitor was coalesced with Chitosan (CS)/Fucoidan (FCD) to form a mucoadhesive nanoparticle formulation with a comparatively lower toxicity profile. This CXB-CS/FCD combination showed remarkable results in terms of reduction in the COX-2 expression among these tumor-associated immune cells. This was accompanied by a reduction of Arginase/Inducible Nitric Oxide Synthase levels, proliferative markers such as IL-6, TGFβ levels, and stemness markers such as CD44 and ALDH, via downregulation of the Jagged-1/Notch signaling. Overall, these cells showed reduced histologic tumor grade, EMT, and metastatic potential, concomitant with lessened immune cell activation, recruitment, and immunocompromised tumor background, with special regard to these features in the CD33+/11b+MDSCs. These important findings support the vital anti-cancer immunotherapy potential of such novel drug preparations in comparison to the traditional CXB therapy, in targeting the pro-tumorigenic MDSCs and exemplifying these mucoadhesive nanocarriers in OSCC treatment (199).

CXB has shown good activity earlier as a part of the oral triple-metronomic chemotherapy in platinum-resistant OC patients. This Phase II trial demonstrated that CXB 200 mg/day BID when combined with erlotinib 150 mg/day OD and methotrexate 9 mg/m2/week had improved progression free survival and OS when compared to those receiving weekly chemotherapy with docetaxel or the best supportive care (200). DAPT was found to inhibit OCC growth in combination with low-dose trixton-100, a cell permeation enhancer, by dysregulating the Notch1/HES1 signaling pathway (201). Ghosh S et al. have portrayed the role of Notch signal activation towards Cisplatin resistance in OSCC stem cells. Contrarily, Notch signal inactivation was associated with a potentially fatal upregulation of the JAK-STAT pathway resulting in increased tumor aggressiveness, stemness, and an unfavorable prognosis. However, stemness was maintained in both the Notch-active and inactive cells, along with the spontaneous coexistence of both the cell states in OSCC. These findings were in support of the antitumor activity of JAK-inhibitors such as Ruxolitinib and Tofacitinib, both of which reduced the hostile tumor proliferation and stemness, in addition to the downregulated Notch-signaling. The addition of γ-secretase inhibitors like LY411575 or RO4929097 helps oral CSCs maintain the Notch-inactive state and following them with the above JAK inhibitors could be a potential therapy option (202). Silencing novel pathways like the FAS-ERK-JAG1-NOTCH1 axis could also result in decreasing the OSCC stemness and the risk of pulmonary metastases, making this a good target for advanced therapies in the future (203).

## 6 Discussion

The review provides a comprehensive overview of stemness influencing factors that are widely spread across cellular processes. The highlighted cross-talks under different sections are of paramount importance in targeting the OSCC stem cells, the very seed of the malignancy that up-holds promising solutions in confronting OSCC (204).

The initial portion of this review illustrates major dysregulated interplays between TFs-miRNA to confer heightened insights on their roles in determining stem cells’ phenotypical characters. The essence of this section is believed to fetch futuristic miRNA-targeted therapeutic strategies in OSCC. One such strategy in targeting the dysregulated TFs-miRNA network is via negating the ectopic expressions of miRNA in the neoplastic cells with the employment of miRNA mimics/inhibitors to impede the levels of oncogene expression (205). For example, the 2017 clinical trial NCT02369198 was based on testing the TargomiR, minicells loaded with miRNA mimics, aimed at targeting EGFR. The mimics were designed to compensate for the loss of the miR-16 family in malignant pleural mesothelioma (206). Of interest in this review, dampening the effect of oncogenic TFs could reduce the stemness features in OSCC. In addition to their utility as a monotherapy in cancer, miRNA mimics have also secured their position in combination therapies with standard chemotherapy agents and RT. miRNA mimetics that interact with the same targets as that of standard therapy may act as adjuvants to reduce the dose and toxicity of these conventional therapies (207, 208).

Additionally, the elucidated TME extrinsic factors in this review provide a plethora of cross-talks that are essential for nurturing and survival of CSCs. This fact opens up an abundant source of targets that may be utilized in encountering the stemness features of OSCC. For instance, inhibitors of Hedgehog and Notch pathways have exhibited substantial advancement in the preliminary stage of clinical trials (209). The translational success of these targets requires a panoramic view, as signaling pathways are complex and follow a nonlinear fashion. The pathways are interconnected with influential cross-talks that regulate various stemness signaling cascades. One such incident could be witnessed in the interplay between PI3K and Notch signaling pathways that lead to the amplification of CSC features and resistance gained towards PI3K inhibitors. Such interactions are often attributed to the observed clinical treatment failures of triple-negative breast cancer (210). The TME interplays also comprehend the underlying mechanism of drug resistance. 5-FU and Paclitaxel were found to have no therapeutic effects over malignant squamous cells due to TGFβ-1 given the development of a quiescent state, especially in the G1 phase of the cell cycle in HNC cells (211).

The review also continues to ascribe various roles of oral microbiota in the acquaintance of stemness in OSCC. The studies presented in this section provide the avenue for future investigation to explore the molecular interaction evinced between commensal microbes in the oral cavity and the premalignant/malignant TME for better treatment approaches in OSCC. In conclusion, this review delivers a broad horizon of stemness governing factors for future research and promising solutions for OSCC treatment challenges.

## Author contributions

SJV, GR, GS, and HA have contributed in conceptualization and literature selection. KaS, MA, SJV, HA, GR, GS, and RH have contributed in manuscript writing and development. KaS, MA, RH, GR, KsS, HB, and PV have contributed in developing schematics in addition to manuscript writing. SSV, DA, MA, RH, KA, FA, IH, HB, and SP have reviewed and revised the manuscript. All authors contributed to the article and approved the submitted version.

## Glossary

**Table:**

OSCC	Oral Squamous Cell Carcinoma
HNC	Head and Neck cancer
HPV	Human papillomavirus
EBV	Epstein–Barr virus
CSC	Cancer Stem Cell
TF	Transcription Factors
TME	Tumor Microenvironment
MSC	Mesenchymal stem cell
EMT	Epithelial to Mesenchymal Transition
ECM	Extracellular Matrix
FCD	Fucoidan
CAF	Cancer-associated fibroblast
GRO-α	Growth-Regulated Oncogene alpha
TGFβ	Transforming Growth Factor β
SMA	Smooth muscle actin
TGFβ	Transforming Growth Factor β
JMJD6	Jumonji domain-containing protein 6
TNF-α	Tumor necrosis factor alpha
TAM	Tumor associated macrophage
OCC	Oral Cancer Cell
OC	Oral Cancer
UPR	Unfolded Protein Reaction
HIF	Hypoxia Inducible Factor
CS	Chitosan
THBS-1	Thrombospondin-1
MMP	Matrix Metalloproteinase
RT	Radiotherapy
Gal-1	Galectin-1
MDSC	Myeloid-Derived Suppressor Cell
OPSCC	Oropharyngeal Squamous Cell Carcinoma
CK19	Cytokeratin 19
CXB	Celecoxib
UPR	Unfolded Protein Response
CBP	cAMP-responsive element binding-binding protein
SATB2	Special AT-rich sequence-binding protein 2
5-FU	5-Fluorouracil
